# Subcutaneous progesterone versus vaginal progesterone for luteal-phase support in frozen-thawed embryo transfer: A cross-sectional study

**DOI:** 10.18502/ijrm.v19i2.8469

**Published:** 2021-02-21

**Authors:** Abbas Aflatoonian, Banafsheh Mohammadi

**Affiliations:** Department of Obstetrics and Gynecology, Research and Clinical Center for Infertility, Yazd Reproductive Sciences Institute, Shahid Sadoughi University of Medical Sciences, Yazd, Iran.

**Keywords:** Progesterone, Subcutaneous, Vaginal, Pregnancy.

## Abstract

**Background:**

Luteal-phase support is a complex and controversial issue in the field of reproductive management.

**Objective:**

To compare the safety and efficacy of low-dose subcutaneous progesterone with the vaginal progesterone for luteal-phase support in patients undergoing rozen-thawed embryo transfer.

**Materials and Methods:**

In this cross-sectional study, information related to 77 women that had frozen-thawed embryo transfer was reviewed. The patients were divided into two groups based on the route of progesterone administration used as a luteal-phase support. When the endometrial thickness reached ≥ 8 mm, in one group progesterone (Prolutex) 25 mg/ daily subcutaneous and in another group, vaginal progesterone (CyclogestⓇ) 400 mg twice or (EndometrinⓇ) 100 mg thrice daily, were administrated and continued until menstruation or in case of clinical pregnancy for 8 wk after the embryo transfer when the fetal heart activity was detected by ultrasonography.

**Results:**

The patient's characteristics were matched and there was no significant difference. The chemical and clinical pregnancy rate was higher in the vaginal progesterone group compared to the prolutex group, but statistically unnoticeable, (40% vs. 29.6%, p = 0.367) and (28% vs. 22.2%, p = 0.581), respectively.

**Conclusion:**

The findings of this study demonstrate that the new subcutaneous progesterone can be a good alternative for intramuscular progesterone in women that dislike and do not accept vaginal formulations as luteal-phase support in assisted reproductive technology.

## 1. Introduction 

Additional embryos obtained through in vitro fertilization (IVF) or intracytoplasmic sperm injection can be stored and transferred in frozen-thawed cycles to prevent the waste of embryo and raises the chance of pregnancy in a single stimulated cycle (1). Various factors such as patient's age, endometrial thickness and pattern, progesterone administration, quality, and the development stage of the embryo, cryopreservation methods affect the factors of pregnancy outcomes (2). Despite the crucial role of progesterone in the luteal phase, based on the route of administration, it has different pharmacological efficacy (3). The different types of progesterone administration include oral, vaginal, oil-based intramuscular (IM) and new aqueous subcutaneous (SC) progesterone. Oral progesterone has poor bioavailability with limited usage in infertility management due to extensive first-pass metabolism in liver. Vaginal progesterone notwithstanding lower circulating levels reaches sufficient endometrial concentration and has good efficiency, but a number of side effects such as vaginal discharge or local irritation, discomfort, and doubt about sufficient absorption has reduced its compliance. Furthermore, because of the apprehensions stemming from different cultural beliefs and personal concerns, many patients avoid the consumption of vaginal progesterone. Beside, oil-based IM progesterone has a fixed dosage but can be really painful, even forming sterile abscesses in some cases (4).

A novel water-soluble SC progesterone (Prolutex) is a good alternative for IM progesterone, it is offered because of equal pharmacokinetic effect to IM P and its additional appropriate self-administration. Although, many studies have proven the safety and efficacy of Prolutex, very few have demonstrated patients' acceptance and opinions about it (5). Prolutex is a synthesis of P and hydroxypropyl-b-cyclodextrin in water that administers SC in a daily dose of 25 or 50 mg. No difference was seen in the endometrial biopsies in the menstrual cycle between the two doses when endogenous progesterone suppressed (6). A study established that the least dose of SC progesterone (25 mg) has equal effect to the daily amount of physiologic of progesterone that produced by the ovary in mid-luteal phase and predecidual changes have seen in all of the endometrium samples (7). Therefore, rather than the 50 or 100 mg doses, we chose the lowest dose in our examination for luteal-phase support (LPS), which demonstrated fewer skin reactions and more safety (8).

The purpose of this study was to compare the safety and efficacy of the low-dose SC Progesterone with the vaginal progesterone for LPS in patients undergoing frozen-thawed embryo transfer (FET).

## 2. Materials and Methods

This cross-sectional study was conducted at the Yazd Reproductive Sciences Institute and the Madar Hospital in Yazd. Data were obtained from the assisted reproductive technology (ART) database over a six months period between March and September 2019. In this study, the medical records of 77 women was retrieved and reviewed.

All women included in this study had previously undergone IVF or intracytoplasmic sperm injection cycles and had embryo cryopreservation. Women aged > 40 yr, with body mass index (BMI) > 30 kg/m${}^{2}$, history of diabetes mellitus and hypertension, severe endometriosis, uterine myomatosis, > 2 implantation failures were excluded from the study. Based on the LPS protocol, the patients were divided into two groups: group I (n = 27) that received Prolutex 25 mg (IBSA Institut Biochimique SA, Switzerland) and group II (n = 50) that used vaginal progesterone CyclogestⓇ (Cox Pharmaceuticals, Barnstaple, UK) or EndometrinⓇ (Ferring Pharmaceuticals Inc., Parsippany, NJ, USA). We used 6 mg/day orally Estradiol valerate (Estradiol Valerate, Aburaihan Co., Tehran, Iran) for Endometrial preparation in both groups from the second day of the menstrual cycle. Ultrasound was done from day 13, endometrial thickness was measured at the maximum diameter in the fundal section.

When the endometrial thickness reached ≥ 8 mm, women in group I received SC progesterone (Prolutex 25 mg; IBSA Institut Biochimique SA, Switzerland) and those in group II received vaginal progesterone (CyclogestⓇ 400 mg twice or EndometrinⓇ 100 mg thrice) daily until negative pregnancy test or for 8 wk after embryo transfer until a fetal heart activity was detected by ultrasound. The embryos were thawed two days after the start of progesterone consumption. Embryo transfer was conducted in cleavage-stage one day after the thawing was performed under ultrasound guidance. Two good-quality embryos (grade A, and B) were transferred to each group using a Cook catheter (Cook Medical, Indiana, USA). A positive pregnancy test was defined as β-hCG > 50 IU/L 14 days after the embryo transfer that was called chemical pregnancy. Secondary outcomes included: Definition of clinical pregnancy is revealing of fetal heart activity in transvaginal ultrasonography 2-3 wk after the positive β-HCG; implantation rate is the ratio of gestational sacs to the number of embryos transferred.

In addition, an abortion was defined as the loss of pregnancy before the 20^th^ wk of gestation while an ongoing pregnancy was considered as a pregnancy continuing beyond the 12${}^{\mathrm{th}}$ wk of gestation.

### Ethical considerations

This study was approved by the Ethics Committee of the Yazd Reproductive Sciences Institute, Shahid Sadoughi University of Medical Sciences, Yazd, Iran (IR.SSU.RSI.REC.1398.027).

### Statistical analysis 

We analyzed the data by using the statistical package for the social science version 26 for windows (SPSS Inc., Chicago, IL, USA). A student's *t* test was apply to assess other variables. We used The Chi-square test for comparition the non-continuous variables. A P-value < 0.05 is statistically significant.

## 3. Results

This study was conducted on 77 women, of which 27 patients were included in the Prolutex group and 50 in the vaginal progestrone group (Table I). No statistically significant difference was observed between the two groups (Table I). The patients were selected from different infertility etiologies and there was no difference between them (Table II). The clinical results showed that chemical and clinical pregnancy rates were higher in the vaginal P group than the prolutex group but the difference was statistically insignificant (40% vs. 29.6%, p = 0.367) and (28% vs. 22.2%, p = 0.581), respectively (Table III).

**Table 1:** Comparison of patient's characteristics between the two groups


**Patients characteristics**	**Prolutex group (n = 27)**	**Vaginal progestrone group (n = 50)**	**P-value**	**CI (95%)**
**Age (yr)**	29.48 ± 4.79	31.30 ± 4.35	0.096	(-3.96-0.32)
**BMI (kg/m${}^{2}$)**	23.73 ± 2.03	23.13 ± 1.66	0.168	(-2.58-1.45)
**Infertility duration (yr)**	5.55 ± 3.35	5.600 ± 2.86	0.951	(-1.49-1.40)
**AMH (ng/ml)**	2.62 ± 1.45	2.61 ± 1.20	0.960	(-0.60-0.63)
**Total embryo **	7.51 ± 4.29	6.18 ± 3.14	0.122	(-0.36-3.04 )
**Endometrial thickness (mm)**	9.75 ± 1.15	9.26 ± 0.85	0.055	(0.37-0.95 )
**Transfer day**	17.3 ± 1.30	16.9 ± 0.88	0.088	(-0.65-0.93)
white<bcol>5</ecol>Data are presented as Mean ± SD, Student *t* test, CI: Confidence interval, AMH: Anti-Mullerian hormone, BMI: Body mass index

**Table 2:** Comparison of different etiologies between the two groups


**Variables **	**MF**	**PCO**	**OF**	**TF**	**Unexplained**	**Mixed**	**P-value**
**Prolutex group**	6 (22)	4 (14)	8 (29)	2 (7)	0 (0)	7 (25)	0.419
**Cyclogest group**	12 (24)	5 (10)	11 (22)	5 (10)	7 (14)	10 (20)	
Data presented as n (%), MF: Male factor, PCO: Polycystic ovary, OF: Ovarian factor, TF: Tubal factor							

**Table 3:** Comparison of pregnancy outcomes between the two groups


**Variables**	**Prolutex group (n = 27)**	**Vaginal progestrone group (n = 50)**	**CI (95%)**	**P-value**
**Implantation rate (%)**	6/54 (11.1)	14/100 (14)	-0.13- 0.76	0.587
**Chemical pregnancy rate (%) **	8 (29.6)	20 (40)	0.23-1.71	0.367
**Clinical pregnancy rate (%) **	6 (22.2)	14 (28)	0.24-2.20	0.581
**Ongoing pregnancy rate (%)**	6 (22.2)	14 (28)	-0.36-0.37	0.581
**Abortion rate (%)**	2 (7.4)	6 (12)	0.31-0.55	0.791
Data are presented as n (%), Chi-squared test, CI: Confidence interval				

## 4. Discussion

The purpose of this study was to evaluate a new progesterone supplementation as LPS in FET cycles, which differs from other available preparations in dosage (25 mg/day) and manner of administration (SC) (4). Our data analysis showed that new SC progesterone resulted in a similar pregnancy outcome when compared with cycles with vaginal progesterone supplementation.

Although, many studies have investigated the effect of different routes of progesterone administration, most of them were directed in fresh IVF cycles. Since there is still no agreement on the method of use, this still remains a controversial issue (7, 9-12). In some literature, LPS has been continued until 10-12 wk of gestation, however, there is a confirmation about withdrawing P on the day of positive pregnancy test or detection of fetal heart beat without increasing the miscarriage rate (9, 10). So, in our study, we administered for 8 wk in pregnant women. Vaisbuch in a web-based universal review showed IM progesterone utilized in 13% of IVF cycles, whereas in North America nearly 60% used alone or with vaginal progesterone (11). Several observational studies showed that the differences in efficacy due to the different forms of progesterone administration in pregnancy likelihood are small (12, 13). So the route of progesterone used for LPS did not affect the live birth rate (14).

Lockwood and coworkers have conducted the first large prospective randomized trial 2014 and compared Prolutex (25 mg SC daily) with progesterone gel Crinone (90 mg intravaginal daily) as an LPS, they showed no significant difference in pregnancy outcomes. The ongoing pregnancy rate per protocol in the Prolutex and Crinone groups was 29.2% and 31.2%, respectively (difference -2.00, 95% CI -9.12-5.13) (7).

Similarly, Baker and coworkers compared Prolutex with vaginal progesterone inserts (Endometrin) 100 mg twice daily in patients undergoing fresh embryo transfer as an LPS. No significant difference was observed in either of the secondary end-points between the two groups (11). Chemical pregnancy (56.4% SC vs 59.0% vaginal; 95% CI -9.5, 4.3), clinical pregnancy (42.6 vs 46.4%; 95% CI -10.8, 3.2).

The results of our study is in line with other investigations showing that the SC aqueous progesterone (Prolutex) 25 mg daily has an equal effect as that of vaginal progesterone. The clinical and ongoing pregnancy rates were 22.2% with Prolutex and 28% with vaginal progesterone (p = 0.581), hence, no significant difference was seen in any of the secondary outcomes, including the implantation rate and abortion.

Although numerous examinations were done for evaluating the reproductive outcomes of all available types of progesterone planning for LPS, limited studies have reflected on patient satisfaction. Only one study has been published so far about the patient's acceptation of SC progesterone with blastocyst transfer in FET cycles; in women that had prior experience of vaginal progesterone, SC progesterone was related with significantly increased receipt, but there was a lack of data about the pregnancy outcomes in this study (5). Vaginal progesterone has different forms, such as capsules, gels, and pessaries. In two studies, patients preferred vaginal gel rather than IM injections as the former is more easier-to-use, more contented, and more rapidly used (15). IM progesterone causes a measurable and fixed serum level but due to some adverse reactions which include pain, local irritation, sterile abscess, and limitation on self-administering it has low patient acceptance (3, 16). The bioavailability of Prolutex with more rapid absorption is comparable to the IM form in serum level concentration and has higher serum levels than vaginal progesterone (8), however, the endometrial levels are higher when progesterone has been used vaginally (7). Prolutex, as mentioned earlier, can be well-tolerated and is not painful.

Therefore, it can be suggested that for those women who do not accept vaginal preparations and prefer constant LPS or dislike vaginal treatments because of social, personal, or medical causes are also worried about the leakage of drugs and are doubtful about the absorption of sufficient dose. In women with vaginal bleeding, use of a vaginal progesterone can be unpleasant (17). All patients had the experience of SC injection that used gonadotrophins in the hyperstimulation cycle (11).

Finally, we remind that a large number of investigations have been registered in the International clinical trial and there is a lack of information about this SC formulation in the field of oocyte donation and FET cycles. In the near future, we expect to face optimal finding in this context (dose and route) that has not been well-defined, from large, well-designed, and multicenter RCTs.

The limitations of this study are: first, restrictions on access to Prolutex in Iran that led to reducing the study's sample size; second, this survey has been done retrospectively. We assume that the longitudinal time frame between the medication and data record and also negative pregnancy tests may negatively influence women's perception and feelings about the drug; therefore, our patient satisfaction was not considered.

## 5. Conclusion 

In this study, we attempted to show that the new SC progesterone formulation is comparable with vaginal progesterone for LPS, there was no statistically significant difference in pregnancy outcomes.

##  Conflict of Interest

The authors have no conflict of interest to declare.
